# 579. Design and Immunogenicity of a Pan-SARS-CoV-2 Synthetic DNA Vaccine

**DOI:** 10.1093/ofid/ofab466.777

**Published:** 2021-12-04

**Authors:** Katherine Schultheis, Charles C Reed, Viviane M Andrade, Richa Kalia, Jared Tur, Blake Schouest, Dustin Elwood, Igor Maricic, Arthur Doan, Zeena Eblimit, Patrick Pezzoli, Dinah Amante, maria yang, Joseph g Fader, Roi Ferrer, David Weiner, J Joseph Kim, Laurent Humeau, Stephanie Ramos, Trevor R F Smith, Kate Broderick

**Affiliations:** 1 INOVIO Pharmaceuticals, Plymouth Meeting, Pennsylvania; 2 Inovio Pharmaceuticals, San Diego, California; 3 Inovio, San Diego, California; 4 INOVIO Pharamceuticals, San Diego, California; 5 Wistar Institute, Philadelphia, Pennsylvania

## Abstract

**Background:**

First-generation COVID-19 vaccines are matched to spike protein of the Wuhan-H1 (WT) strain. Convalescent and vaccinee samples show reduced neutralization of SARS-CoV-2 variants of concern (VOC). Next generation DNA vaccines could be matched to single variants or synthetically designed for broader coverage of multiple VOCs.

**Methods:**

The synthetic consensus (SynCon®) sequence for INO-4802 SARS-CoV-2 spike with focused RBD changes and dual proline mutations was codon-optimized (Figure 1). Sequences for wild-type (pWT) and B.1.351 (pB.1.351) were similarly optimized. Immunogenicity was evaluated in BALB/c mice. Pre-clinical efficacy was assessed in the Syrian Hamster model.

Figure 1. Design Strategy for INO-4802

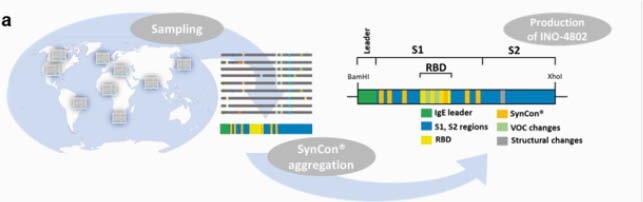

**Results:**

INO-4802 induced potent neutralizing antibody responses against WT, B.1.1.7, P.1, and B.1.351 VOC in a murine model. pWT vaccinated animals showed a 3-fold reduction in mean neutralizing ID50 for the B.1.351 pseudotyped virus. INO-4802 immunized animals had significantly higher (p = 0.0408) neutralizing capacity (mean ID50 816.16). ID50 of pB.1.351 serum was reduced 7-fold for B.1.1.7 and significantly lower (p = 0.0068) than INO-4802 (317.44). INO-4802 neutralized WT (548.28) comparable to pWT. INO-4802 also neutralized P.1 (1026.6) (Figure 2). pWT, pB.1.351 or INO-4802 induced similar T-cell responses against all variants. INO-4802 skewed towards a TH1-response. All hamsters vaccinated with INO-4802 or pB.1.351 were protected from weight loss after B.1.351 live virus challenge. 4/6 pWT immunized hamsters were completely protected. pWT immunized hamsters neutralized WT (1090) but not B.1.351 (39.16). INO-4802 neutralized both WT (672.2) and B.1.351 (1121) (Figure 3). We observed higher increase of binding titers following heterologous boost with INO-4802 (3.6 – 4.4 log2-fold change) than homologous boost with pWT (2.0 – 2.4 log2 fold change) (Figure 4).

Figure 2. INO-4802 Induces Functional Humoral Immune Response Against SARS-CoV-2 Variants of Concern

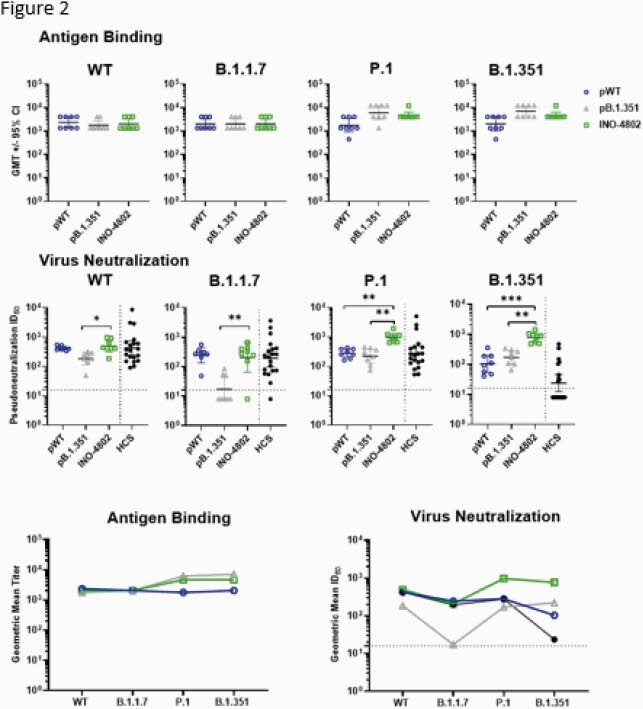

Figure 3. INO-4802 Protects Hamsters Against Challenge With B.1.351 Live Virus

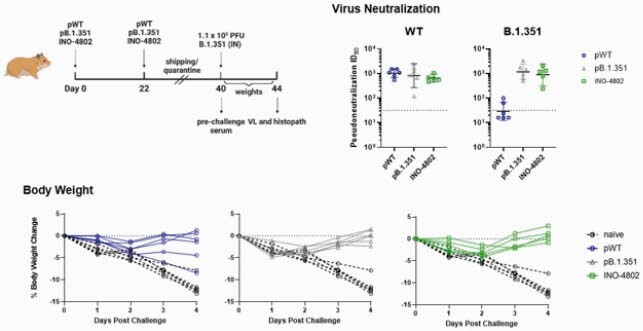

Figure 4. Heterologous Boost with INO-4802 Induces Humoral Immune Response Against SARS-CoV-2 Variants

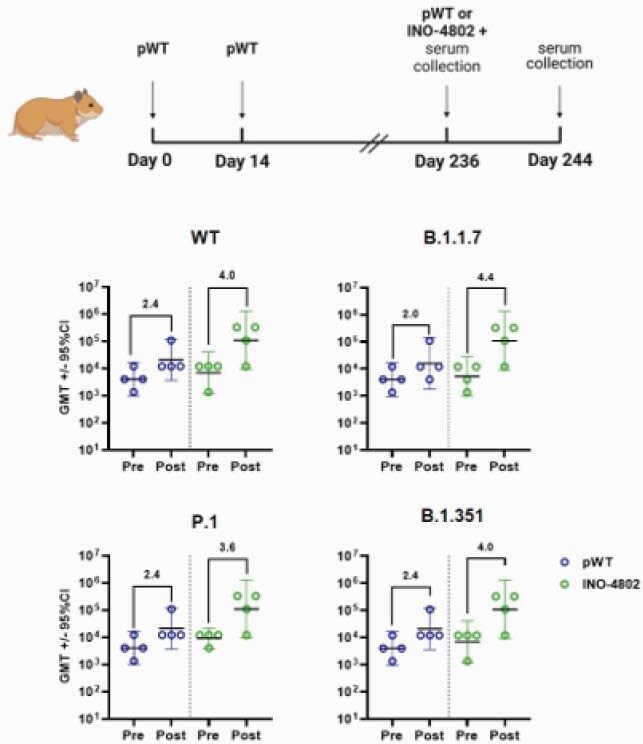

**Conclusion:**

Vaccines matching single VOCs, like pB.1.351 and pWT, elicit responses against the matched antigen but have reduced cross-reactivity. Presenting a pan-SARS-CoV-2 approach, INO-4802 may offer substantial advantages in terms of cross-strain protection, reduced susceptibility to escape mutants and non-restricted geographical use.

**Disclosures:**

**Katherine Schultheis, MSc**, **Inovio Pharmaceuticals** (Employee) **Charles C. Reed, PhD**, **Inovio Pharmaceuticals** (Employee, Shareholder) **Viviane M. Andrade, PhD**, **Inovio Pharmaceuticals Inc.** (Employee) **Richa Kalia, MS**, **Inovio Pharmaceuticals** (Employee, Other Financial or Material Support, I have stock options with Inovio Pharmaceuticals as an employee.) **Jared Tur, PhD**, **Inovio** (Employee) **Blake Schouest, PhD**, **Inovio Pharmaceuticals** (Employee) **Dustin Elwood, PhD**, **Inovio Pharmaceuticals** (Employee) **Arthur Doan, n/a**, **Inovio** (Employee) **Patrick Pezzoli, BS**, **Inovio** (Employee) **Dinah Amante, BS**, **Inovio** (Employee) **David Weiner, PhD**, **Inovio** (Board Member, Grant/Research Support, Shareholder, I serve on the SAB in addition to the above activities) **J Joseph Kim, PhD**, **Inovio** (Employee) **Laurent Humeau, PhD**, **Inovio Pharmaceuticals** (Employee) **Stephanie Ramos, PhD**, **Inovio Pharmaceuticals** (Employee) **Trevor R. F. Smith, PhD**, **Inovio** (Employee, Shareholder) **Kate Broderick, PhD**, **Inovio** (Employee).

